# On the Trail of the Longest Plant RNA Virus: Citrus Tristeza Virus

**DOI:** 10.3390/v17040508

**Published:** 2025-03-31

**Authors:** Moshe Bar-Joseph

**Affiliations:** The S. Tolkowsky Laboratory, Department of Plant Pathology & Weed Research, ARO-The Volcani Center, P.O. Box 15159, Rishon Lezion 7528809, Israel; mbjoseph@gmail.com

**Keywords:** closterovirus, virus isolation, ELISA, seedling yellows tristeza, mild tristeza isolates, VT strain of CTV, aphid transmission, pathogen suppression, double-stranded RNA, defective RNA, transgenic resistance, tristeza history

## Abstract

The devastating tristeza epidemic swept through South American citrus groves in the 1930s and subsequently spread to most citrus-growing regions worldwide, causing varying degrees of damage and prompting significant changes in citrus cultivation practices. The causal agent of the disease, citrus tristeza virus (CTV), belongs to the genus *Closterovirus* in the family *Closteroviridae*. CTV virions are approximately two microns long and possess the largest known positive-strand RNA genome in plants, spanning 19.3 kb. The history of tristeza disease and CTV’s molecular biology and taxonomic relationships have been extensively reviewed in the scientific literature. This paper primarily focuses on the author’s personal experiences with tristeza disease and its causal agent over the past six decades. The journey began during a period when biological indexing was the primary diagnostic tool. It later progressed through the isolation of purified CTV particles, which served as a practical diagnostic tool for CTV suppression efforts in Israel during the 1970s. However, biological indexing was first replaced by electron microscopy, followed by ELISA procedures; both were eventually abandoned after it was discovered that many ELISA-positive infections were caused by symptomless CTV isolates, even on trees grafted onto sour orange rootstocks. In retrospect, my work on CTV can be categorized into three main phases. It began with the biological phase, inherited from earlier generations of citrus virologists, followed by the isolation and partial characterization of CTV virions, and culminated in the genomic era. While we live in an age of remarkable biotechnological achievements, my recommendation for future CTV research is to integrate both biological and genomic approaches rather than viewing them as mutually exclusive. This is particularly important for economically significant pathogens such as CTV, which should be studied continuously as both biological agents and molecular pathogens.

## 1. Introduction

This paper reflects on the professional insights gained from my long association with citrus trees, as well as my research and practical work on viruses and virus-like diseases affecting citrus. My primary focus has been on the citrus tristeza virus (CTV), a member of the genus *Closterovirus* in the family *Closteroviridae*, which is considered one of the top ten most important plant pathogens globally [1]. Extensive information on tristeza disease and its causal agent CTV—recognized for having the largest known single-stranded positive-sense RNA genome [2,3,4,5,6,7,8,9]—as well as its taxonomy [10,11,12,13,14,15], has been documented in the literature.

In this largely autobiographical paper, a disproportionate number of the references will be self-citations, reflecting my personal contributions over the decades. Additionally, some significant publications on CTV authored by close colleagues and friends may be missing. This imbalance is unintentional and does not reflect the relative importance of the uncited works.

Citrus pathology and citrus horticulture are closely intertwined, as demonstrated by the significant shift in citriculture practices in response to the epidemic of Phytophthora gummosis. It was noted first in Madeira Island in 1832 and spread to citrus areas close to ports of the Mediterranean basin, causing havoc in the luxurious industry of Vitamin C supply to sailors on the route to the Far East. The gummosis problem was elegantly solved in Spain by grafting the sensitive trees onto tolerant sour orange rootstocks [4]. The practice was rapidly adapted throughout the gummosis-affected areas and far beyond, resulting in productive grafted citrus groves. Surprisingly, however, in areas like Australia, grafting onto sour orange rootstocks failed [16]. The reason behind this initially mysterious problem was, in later years, realized as the result of the wide presence in these areas of the citrus pathogen, which was decimating citrus trees grafted on sour orange rootstocks [4,17]. Later, the transfer of CTV-infected budwood from South Africa to South America, where the most effective CTV vector, *Aphis citricidus*, was already present, led to the rapid spread of decline. The disease was first considered a root rot problem and was later renamed Tristeza (sadness in Portuguese and Spanish) in South America [4,18,19].

Interestingly, discussions on the manifestation of tristeza disease often overlook the significant damage caused by CTV to non-grafted sour lime trees—whose fruits were used in the Gold Coast (Ghana) soft drink industry [20]. This damage coincided with epidemics affecting groves grafted onto sour orange rootstocks [4]. The vein-clearing symptom of CTV on sour lime seedlings, first observed in Africa, soon became the primary diagnostic tool for tristeza disease [21].

The tristeza-induced quick decline crisis led to the abandonment of sour orange rootstock and prompted the adoption of CTV-tolerant rootstocks, enabling the continued expansion of citrus production in CTV-affected areas [4].

## 2. My First Encounter with CTV

I came to know the term “tristeza” in 1956 at the agricultural school Mikveh-Israel while tasked with organizing an exhibition of exotic citrus fruits from the school’s renowned citrus variety collection. Working at the collection, I had the privilege of meeting Prof. I. Reichert, considered the father of Israeli phytopathology, and his assistant, Mr. A. Bental, who conducted CTV indexing on the collection trees. Their work revealed several exotic varieties in the collection were infected with CTV [22]. Their findings underscored the critical importance of stringent phytosanitary measures and remained relevant throughout my career. In my numerous visits to farms and experimental stations abroad, I was often tempted to acquire interesting, non-patented citrus varieties. As a plant pathologist, however, I was always mindful of the risks of the spread of diseases.

**Graduate studies in plant virology** In 1964, I began my Master’s studies in genetics and plant pathology at the Hebrew University, Jerusalem and Rehovoth. Plant virology has been largely driven by the numerous breakthroughs stemming from studies on tobacco mosaic virus (TMV). TMV was the first pathogen to be classified as a unique biological entity later named a virus. TMV was to be the first virus to crystallize and the first virus observed using electron microscopy (EM). It was also the first virus to be separated into protein and RNA components and the first to demonstrate that its genome is solely RNA based [23,24]. Earlier to these developments, Holmes’ [25] report of local lesion reaction of TMV on certain plant species facilitated the quantification of the virus, while McKinney [26] observed cross-protection between TMV isolates. Over time, TMV became the first plant virus to be sequenced [27] and the first plant virus to be transformed into an infectious vector [28].

Research on plant viruses has revealed significant variation in transmission modes, host ranges, symptom intensities, and virus accumulation levels in infected tissues. For instance, the concentration of TMV virions in an infected tobacco leaf was unusually high compared to most other viral infections.

Contrary to the convenience of mechanical transmission of TMV, only a few citrus viruses could be transmitted mechanically, and those transmissible had concentrations in infected citrus hosts that were much lower than those of TMV. These limitations were frequently highlighted in virological journals of the time. The vast majority of plant virus publications during that period focused on TMV and half a dozen other mechanically transmissible plant viruses that also reached high titers in infected host plants. In contrast, many important plant viruses, including those like CTV, which were responsible for significant plant diseases, were rarely mentioned in publications of the prestigious virology journals of that era. The situation was even more complex for certain graft-transmitted citrus diseases suspected to be caused by viruses, such as citrus Impietratura. These diseases could not be transmitted mechanically, and EM observations of infected tissue failed to detect any known form of virus particles (Cohen Y. and Bar-Joseph, M., unpublished).

In 1967, I started working as a Ph.D. candidate at the Virus Laboratory, Plant Protection Institute, Volcani Center, Bet Dagan, Israel, under the supervision of the late Prof. Gad Loebenstein. The thesis dealt with the isolation and partial characterization of the thread-like particles (TLPs) noted earlier by Kitajima et al. in Brazil [29] associated with CTV infections. The research was mostly funded by the Israeli citrus industry, which, at that time, was a major contributor to the local economy. The tristeza disease was considered the greatest threat to local production since most citrus orchards were grafted onto the highly CTV-sensitive sour orange rootstocks.

**The Paradox of plant virology success** Interestingly, at the time of my thesis work, there were two plant virology units at the Volcani Institute, which employed about ten researchers. Similar levels of plant virology staffing were common at that time in other research centers of the world, including the John Innes Institute (JII) in Norwich, UK, where I spent my postdoctoral year in 1972–1973. The JII continues to be among the highest-ranking plant biology research centers; however, its plant virus research unit has been drastically reduced, reflecting a broader trend across most other plant science centers. This paradox arises mainly from the fact that plant virology has successfully eliminated many major plant virus disease problems, especially of vegetatively propagated crops. Hence, the funding for ongoing plant virus research has been considerably reduced, making it increasingly difficult to recruit replacements for plant virology positions.

A perfect example of the great success story of citrus virology is illustrated by the elimination of citrus Impietratura [30,31] and citrus psorosis virus (CPV) [32,33] diseases from Israeli citrus groves. In the past, Impietratura, named in Hebrew as ‘Avenet’ and ‘Hajar’ in Arabic, both names with similar meanings to Impietratura (rock in Italian), was one of the most common and problematic citrus virus diseases in local citrus groves.

More than ten years ago, following the developments of next-generation sequencing (NGS), allowing the diagnosis and genomic characterization of unknown or difficult-to-extract viruses [34], I considered applying the method to study the nature of the RNA molecules causing the citrus Impietratura disease. However, my citrus virus collection was discarded following retirement in 2004; I asked Israeli citrus extension friends to assist me in locating trees showing Impietratura symptoms. To my surprise, none of them could find a single Impietratura-diseased tree in the commercial orchards they regularly visit and inspect.

What a change and contrast to the time I started working on Impietratura, about half a century earlier, when Impietratura-diseased trees were often present in most old clone grapefruit and Valencia groves in Israel. The elimination of Impietratura from the present-day commercial citrus groves is not unique to either the particular citrus disease or Israeli citrus. Along with Impietratura, several other non-vectored citrus virus diseases have been successfully eliminated. The success of essentially eliminating these citrus disease problems was the result of technological improvements in virus elimination from infected budwood and the mandatory adoption of strict regulatory rules enforcing the use of disease-free budwood for citrus tree propagation [35]. In retrospect, my research and development work in citrus virology comprised three main phases. It began with the biological phase, inherited from earlier generations of citrus virologists, followed by the isolation of virions, which ultimately led to the genomic era.

## 3. Phase 1: Reliance on Biological Procedures for Disease Identification and Disease-Free Budwood Production

Shortly after starting my PhD work, Mr. Arieh Bental, the person in charge of citrus budwood sanitation at the Virus Laboratory, passed away, and his task of biological indexing of certified citrus trees was transferred to me. We used biological indexing for testing Psorosis, Exocortis, and Tristeza, [21,35] in Oroblanco, Star Ruby, and Minneola tangelo introduced from the USA and later widely planted as replacements for local Shamouty oranges groves. Other varieties tested and released were Clementine lines from Corsica and a series of easy-peeling mandarin hybrids developed by Drs. Spiegel and Aliza Vardi, the Volcani Institute citrus breeders.

Our indexing work revealed the association of psorosis-like leaf symptoms with Impietratura-diseased trees, allowing concomitantly indexing and elimination of both psorosis and Impietratura infections [36].

**Adapting shoot tip grafting to produce disease-free budwood Our** indexing work on local citrus sources revealed numerous viroid infections [35]. In 1975, Dr. Navarro’s laboratory in Valencia, Spain, kindly introduced me and later Dr. Aliza Vardi to a short practical training on the shoot tip grafting (STG) method [37]. Upon our return, we applied STG and indexing techniques to establish a large collection of disease-free local citrus varieties and subtypes. My lab maintained these plants for nearly two decades. Eventually, our disease-free stock became the primary foundation for the centralized budwood distribution facility at the Gilat Research Station, Israel.

## 4. Phase 2: Development and Adoption of Novel Technologies for Rapid and Large-Scale Diagnosis of CTV and Other Plant Disease Agents

For almost three years during my PhD work, all efforts to purify TLP were a continuous failure. In the absence of mechanical transmission of CTV, it was difficult to quantify the purification results, and the only available route was using EM to count TLPs. The main limitation of EM was its inability to assess the results of the procedure until the final purification step since the CTV TLPs were difficult to observe at the intermediate purification stages. I was lucky to be assisted by Mr. Yaacov Cohen, the virology unit electron microscopist. On average, we performed at least three TLP purification attempts per week, starting with the peeling of bark from infected sour lime or *C. macrophylla* shoots. This was followed by macerating the bark using liquid nitrogen, suspension of the frozen powder in a buffer, clarification by low-speed centrifugation, and virus pelleting using ultracentrifugation. The procedure was completed by placing the pellet expected to contain the TLP for overnight resuspension solution in the cold room. By the next morning, a few drops from the preparation were used for EM observation. The results were continuously meager, with few, if any, TLPs observable on the grids. Endless variations of the procedure were performed, and the results continued to be discouraging. In retrospect, it took me years to appreciate Prof. Loebenstein’s patience with my inability to deliver my PhD task of isolating TLPs on time. Only by adopting a modified PEG procedure [38] for TLP isolation did we obtain EM pictures showing concentrated purified TLPs [39], which were a pleasant reward to the endless previous frustration. Unfortunately, despite the availability of purified TLPs, we did not continue with the original plan of antisera production, mainly because we realized from experience with the related carnation necrotic (yellow) fleck virus (CNFV) [40] that the serological procedures available at that time were not sensitive for practical CTV diagnosis.

## 5. Natural Spread and the CTV Suppression Program in Israel

Coincidentally, around the time we completed the task of isolating the CTV virions [39], the citrus extension specialists Mr. S. Ashkenazi and Mr. Yair Oren brought to our attention the finding of a couple of declining Valencia trees grafted onto sour orange rootstocks in the village of Hibbat Zion. Using the newly developed PEG isolation and electron microscopy (PEG-EM) procedure [41], we regularly observed the TLPs in the tissue extracts of the declining trees, confirming CTV infection. Trees in the affected grove originated from a certified nursery, suggesting the country’s first cases of natural CTV spreading. The PEG-EM procedure, which could be completed within 2–3 days, proved invaluable as a supplement to traditional biological indexing, which required 1–3 months to complete. This method enabled us to rapidly screen a nearby symptomless Clementine grove close to the declining Valencia trees, with a more than a 10% infection rate. Consequently, the entire grove was discarded to prevent further transmission [42].

A subsequent visual inspection identified another declining Valencia tree in a plot owned by Mr. J. Vizer, located about half a kilometer south of the initial infections. Tissue from this tree yielded the CTV-VT isolate, which was later found to be highly transmissible by different melon aphid *Aphis gossypii* populations [43,44] and to induce a severe seedling yellows (SY) reaction [43,45,46].

These alarming findings prompted the Israeli citrus industry to initiate a coordinated CTV suppression program to eliminate infection foci in the Hibbat Zion area, which included over 100,000 trees across approximately 250 commercial plots and home gardens in central Israel.

Three governmental bodies participated in this ambitious program: **Citrus Extension Services** was responsible for sample collection; **our laboratory** conducted diagnostic testing; and **Plant Protection and Inspection Services (PPI)** managed the elimination of infected trees and handled compensation for disposed groves with infection rates exceeding 10%.

The diagnostic procedure combined grafting multiple (five to eight) sample buds from suspected trees onto sour lime plants. Once an indicator plant showed CTV symptoms, all budwood source trees grafted onto the diseased indicator were retested using PEG-EM. Details of Israel’s CTV eradication efforts have been previously reported [3].

**The introduction of ELISA for CTV diagnosis** Plant virus detection using ELISA was practiced first at the East Malling Station, England [47]. Learning about this development, we invited Dr. Michael Clark for a short visit and realized the advantages of using ELISA for our large-scale CTV diagnostic needs [48]. As mentioned earlier, producing CTV antisera was not considered a priority, mainly because in the pre-ELISA period, serological detection of Closterovirus, like CNFV [40], necessitated CTV antigen concentration unavailable in CTV-infected samples. Luckily, the Floridian team was more foresighted [49] and kindly provided us with their rabbit antibodies for the early stages of ELISA development. Later, we developed large amounts of polyclonal [50,51] and monoclonal antibodies [52]. ELISA allowed an almost tenfold increase in the number of tested trees, reaching about one million ELISA tests between the summers of 1979 and 1980 [3].

Being the first to use ELISA in Israel, our lab became the training ground for other virology and plant pathology units of the Volcani Institute. We also trained the virus units of two major Local Hospitals and the Veterinary Virology lab at the Faculty of Agriculture.

**The Dangers of Assisting Advanced Technologies** I learned a crucial lesson about the risks of working with and adapting advanced technologies to meet human laboratory needs. This experience occurred when a friend from a local hospital gave me 30 blood samples to develop an ELISA kit for one of his antigens. Upon receiving the samples, I immediately stored them in the department’s cold room.

Shortly afterward, I received a call from the brother of my lab assistant, informing me that she had been hospitalized with meningitis. He asked if we had been working with human pathogens. My immediate response was that we had only been working with plant pathogens. However, after the call, I remembered the blood samples in the cold room. I was overwhelmed with panic for several days, fearing the worst. Thankfully, my assistant made a full recovery.

This incident was a wake-up call, and I solemnly promised myself that I would never again bring anything into the lab for testing unrelated to plants.

## 6. Short-Sightedness Regarding the Commercialization of ELISA for Plant Viruses

In 1980, just before leaving for a sabbatical in the USA, I was visited by Dr. Chet Sutula, the founder of Agdia Inc. (Elkhart, IN, USA), a supplier of ELISA reagents for plant pathogen detection. I must admit, at the time, I did not believe there was a viable business opportunity for plant viruses in the ELISA market.

In our work, the calculated cost of an ELISA test was about ten cents per sample. I could not comprehend how kits could be sold at the high prices required to make a profit, especially when plant virus laboratories were freely exchanging, at that time, milliliters of sera. My shortsightedness became apparent a few years later when I needed an ELISA kit to study citrus vein enation disease [53]. Dr. Sutula kindly provided me with a free ELISA kit. However, subsequent purchases of other plant virus kits were far more expensive than I had initially anticipated.

## 7. Establishing a Collection of Israeli CTV Isolates

The introduction of ELISA for CTV diagnosis in the late 1970s revolutionized the scale and speed of tristeza testing. ELISA’s high sensitivity enabled extensive disease surveillance, including rapid countrywide surveys of CTV infections. These surveys involved pooling samples from five trees collected from thousands of citrus groves, each covering 1–2 hectares, and fruit samples obtained from distant packing houses [3]. The latter used the residual fruit peduncles for testing [54]. These surveys showed that CTV was present far beyond the initially identified foci, providing a clearer understanding of its distribution and incidence [3].

Using ELISA, we identified significant variability among CTV isolates. Interestingly, the variations in ELISA titers were not due to differential reactions of isolates with the polyclonal antibodies but rather caused by the uneven distribution of different virus isolates within the infected trees. The severe seedling yellows (SY) variants were the most challenging isolates to detect, particularly the Mor-T isolates [55]. In contrast, trees infected with mild, symptomless CTV-VT isolates were considerably easily identified as CTV-positive [45]. Samples from a large Shamouti grove near the Mikveh-Israel citrus collection were among the first to show this pattern. In later years, the prevalence of mild isolates increased, raising questions about the horticultural and economic value of eradication efforts.

An intriguing question emerged: Why did the CTV situation in Israel differ from the experiences in Argentina and Brazil, where entire citrus groves on sour orange rootstocks collapsed? In Israel, grove destruction caused by natural CTV infections was rare. Most ELISA-positive groves exhibited symptomless infections, leading to growers opposing tree removal. They challenged the rationale for the continued suppression of ELISA-positive trees, claiming that the trees were healthy and productive despite being ELISA-positive. Interestingly, when graft-inoculated onto sour orange seedlings, most mild isolates showed behavior resembling the SY recovery phenomena of CTV-VT isolates [46]. Differences in climatic conditions between the Mediterranean and South American citrus regions seem unlikely to contribute to these observed variations. However, the abundance of inarched citrus trees in Israel at that time may have been a key factor. These trees, with substantial portions of their original sweet lemon rootstocks still alive, provided a degree of tolerance to CTV infection, unlike sour oranges. This tolerance likely prevented the rapid decline in CTV-VT-SY-infected trees and may have facilitated the spread of non-SY isolates of CTV-VT.

## 8. Molecular Advances in CTV Characterization

For my PhD thesis, the characterization of CTV included analysis of the major CTV coat protein (CP) size with PAGE-SDS [56], identical to the calculated protein product of the major CP sequence coding p25 [57].

During my postdoctoral work at JII, I applied the challenging experience of CTV purification to purify a closely related virus, beet yellows virus (BYV) [58]. This task was particularly challenging, as a former JII student assigned to BYV purification had been reassigned after two years of unsuccessful attempts to isolate BYV to a simpler project, which allowed him to complete his thesis within the allotted time.

The CTV and subsequent BYV characterization work at JII, conducted in collaboration with Dr. Roger Hull, led us to propose the classification of CTV and BYV into a new virus group, which we named *Closterovirus* [11,58].

After returning from JII, I became interested in characterizing another Closterovirus, carnation necrotic fleck virus (CNFV) [40]. Unlike CTV, CNFV had a higher concentration of virions in necrotic carnation leaf tissue, and the virus was significantly more stable. Electron microscopy revealed large aggregates of apparently degraded Closterovirus particles, which turned out to be normal by tilting the grids holding the thin sections of tissue [59].

## 9. Double-Stranded RNAs (dsRNAs)

During a sabbatical year (1980–1981) at the Department of Plant Pathology, University of California, Riverside, I continued the CTV characterization [60] and production of antibodies to CTV, which later served the Californian citrus industry for many years. Shortly after my arrival, I had the privilege to cooperate with Dr. Allen Dodds, a newcomer to the department equipped with an effective technology for isolating double-stranded RNA (dsRNA) molecules from tissues infected with various viruses [61]. The stability of dsRNA molecules and the observed variation in their polyacrylamide gel electrophoresis (PAGE) profiles suggested their potential use for CTV diagnosis [62].

Interestingly, just a few years prior to these dsRNA studies, there were still publications that questioned whether dsRNA molecules truly existed or were merely artifacts of phenolic extraction. This debate was elegantly resolved by Derrick et al. [63], who demonstrated the presence of dsRNA molecules in TMV-infected tissues using serologically specific electron microscopy.

Compared to the challenges of isolating CTV virions, dsRNA extraction was significantly simpler and more reproducible. This prompted investigating whether other Closteroviruses—still limited in number at the time—exhibited similar dsRNA profiles. Indeed, plants infected with different Closterovirus species showed considerable similarities in their dsRNA profiles [64].

Two notable mistakes in my dsRNA work are worth mentioning.

The first involved the failing attempt to ligate and clone the abundant short CARNA 5 dsRNAs from naturally infected *Nicotiana glauca* [65] directly into replicating dsDNA plasmids. All our efforts were negative and never published.

Another mistake, the misidentification of the translation product of the CTV p23 dsRNA molecules, did make it to publication: The work was conducted by an excellent postdoc who developed an elegant method for easy and rapid isolation of dsRNA fragments from gels [66]. The isolated dsRNA fragments served as templates for cell-free protein translation. The small and abundant dsRNA molecule, which was later identified to correspond with the p23 subgenomic RNA, was translated into a polypeptide. PAGE runs of the translation product suggested a size similar to the CTV major coat protein and which appeared to precipitate with polyclonal antibodies to CTV [67]. However, this reaction was later found nonspecific, resulting from the sticky nature of the p23 translation product.

In later years, dsRNA molecules associated with *Closteroviridae* became invaluable for cloning and characterizing other members of the *Closteroviridae* family. These studies bypassed traditional virus isolation procedures, using dsRNA extracts as templates for cDNA cloning and genomic characterization [14].

## 10. Cloning CTV cDNA

After returning from UC Riverside, Dr. Dawson’s work on the TMV infectious clone [28] led me to enroll Dr. Aryeh Rosner, formerly in the biotechnology industry, into our CTV group. We cooperated with the late Dr. Irit Ginzburg from the Weizmann Institute and successfully cloned several cDNA fragments of the CTV-VT strain [68]. One fragment, which hybridized specifically only with extracts of CTV-infected plants, proved particularly valuable. It reacted with CTV-VT but failed to recognize the CTV-HT isolate, the sequence of which was noted later to be closely similar to the Floridian T36 isolate [69]. These hybridization results were the first molecular indication of significant genomic divergence between CTV isolates, later categorized into distinct strains.

Using this ability to differentiate between two closely related CTV isolates based on their reactions with the specific CTV–VT probe, we tasked a summer student with determining whether the HT isolate could cross-protect *C. macrophylla* against superinfection by the VT isolate. The experiment was straightforward but yielded perplexing results: as expected, the extracts from the HT isolate infected plants were not initially recognized by the CTV-VT cDNA probe. Surprisingly, shortly after the challenge inoculation, the signals of the superinfected VT isolate were detected in the HT-infected plants, just as in the CTV-VT-challenged control plants. These results were not surprising since earlier practical cross-protection tests indicated that cross-protection occurs only with some mild isolates [70,71]. Interestingly, in later years, Dr. Folimonova, working in Dr. Dawson’s lab, demonstrated that cross-protection occurs only between CTV isolates belonging to the same strain [72,73]. In contrast, HT and VT belonged to different CTV strains [71]. Indeed, our sour orange seedling plants infected with non-seedling yellows (nSY) isolates of CTV-VT were effectively protected against superinfection by the severe SY-CTV-VT isolates [46,74]. However, the practical application of the mild CTV-VT isolates in our case was complicated due to the association of some of the nSY-CTV-VT isolates with severe stem pitting on Oroblanco and Star Ruby grapefruit trees [74]. We revealed a phenomenon when symptomless sweet orange trees infected by non-SY-CTV-VT isolates were top-grafted with healthy budwood of the two stem-pitting sensitive varieties mentioned above.

By 1986, the CTV suppression program had effectively ceased in most areas, except for a single case of spread toward newly planted citrus in the Besor region of the Northern Negev, where suppression efforts continued into the early 1990s. The cessation of the program was deemed necessary, as most ELISA-positive samples were mild VT isolates that did not cause a decline in trees on sour orange rootstock.

Considering the outcome of Israel’s costly 16-year-long suppression program, it can be concluded that the program was largely effective in preventing the major spread of decline-causing isolates. In later years, the program’s closure was justified by the realization that most ELISA-positive trees were infected with symptomless CTV-VT isolates when grown on the sensitive sour orange rootstock. Moreover, these mild CTV-VT isolates seemingly provided protection against superinfection by decline-causing CTV-VT isolates [46]. Thus, the CTV suppression efforts and the supply of disease-free budwood contributed significantly to reducing CTV damage in Israeli citriculture.

## 11. Genetic Analysis of the Coat Protein of Israeli CTV Isolates

Shortly after the Floridian publication of the CTV-T36 coat protein (CP) gene sequence [57], we developed primers for the analysis of the major CTV coat protein genes of the local collection of CTV isolates [75]. Surprisingly, the majority of the isolates, including both severe and mild types, either transmissible or nontransmissible by the local major vector, *Aphis gossypii*, and from different regions in Israel, were mostly genetically closely similar to the CTV-VT type isolate and hence grouped as belonging to the VT strain. Two isolates were notable exceptions. One was derived from an old introduction of CTV-infected Meyer lemon [76], and the other was the local HT isolate that resembled, as mentioned before, the Floridian T36 isolate. The HT isolate had been obtained from a tree showing a decline in Kfar Hayim, suggesting an illegal introduction of diseased budwood. Monoclonal antibodies prepared against the CTV-VT type isolate confirmed the dominance of the VT strain isolates in our CTV isolate collection [52].

## 12. Oligonucleotides and PCR

We regularly used radioactive cDNA fragments of the VT strain for CTV detection [69] and radiolabeled synthetic oligonucleotides (ONs) corresponding to the avocado sunblotch viroid (ASBVd) genome [77] for ASBVd detection [78,79]. The ONs were also applied to detect genetic differences between the type strain isolates of TMV, which we collected under license from a dozen laboratories worldwide to analyze TMV genetic stability. Using two 21-base ON probes, we found that two out of a dozen TMV-type isolates obtained from different geographic regions were not recognized by one of these probes, which was synthesized based on the published genome of TMV-type strain (Bar-Joseph, unpublished). Further work with ONs showed that tobacco transgenes harboring the 21-base ON sequence corresponding to the TMV coat protein failed to provide transgenic protection against TMV (Sadot, E., unpublished MSc thesis).

Interest in the polymerase chain reaction (PCR) was initiated in our lab in the early 1990s, at the pre-automation stage. In those early experiments, Dr Adi Wexler employed manual transfer of PCR reaction tubes through heating and cooling baths. The routine dsRNA analyses of CTV and other virus-infected plants resulted in the accumulation of large quantities of dsRNA molecules, which we, as mentioned above, already attempted to use as templates for dsDNA synthesis.

With the new PCR technology, we devised a novel method where a 20-mer polylinker ONs (OnA) was enzymatically ligated to the 3′ ends of the template dsRNA strands, followed by the use of a second oligonucleotide (OnB) complementary to the ligated primer for cDNA synthesis on the chimeric RNA-OnA molecules. The cDNAs synthesized on the two RNA strands were annealed and extended using Taq polymerase, then amplified by PCR with the primer OnB. The resulting DNA was enzymatically restricted and cloned into a pBluescript vector.

This method [79] enabled the development of a range of cDNA clones from the dsRNAs of banana plants naturally infected by cucumber mosaic virus and citrus infected with CTV [79,80]. A modification of the ligation method was also adopted for viroid cloning [81]. The method’s main advantage was allowing the rapid sequencing of the 5′ and 3′ ends of dsRNA fragments.

This procedure, which closely resembles the oligonucleotide ligation and amplification technologies later adjusted for NGS [34,80], was published in *Methods in Molecular and Cellular Biology* in the early 1990s [79]. Unfortunately, the journal ceased to exist shortly after, leaving our work largely unrecognized and probably missing an opportunity to establish a probable priority for some NGS-ligation-related steps [82].

## 13. Defective RNAs and the CTV-VT Complete Genome

Early in Dr. Munir Mawassi’s PhD work in my lab, we resumed efforts to clone and sequence the CTV-VT cDNA. Using the 3′ end ONa ligation method described above [79], a 2.4 Kbp cDNA fragment specific to the CTV-VT isolate was cloned and sequenced. Repeated attempts to extend the cDNA of the CTV genomic using the 2.4 Kbp-derived specific primers yielded consistently only short cDNA that did not allow extension of the large CTV genomic RNA. The problem stubbornly persisted for a couple of years until we learned that Dr. Dawson’s lab in Florida had nearly completed sequencing the Florida CTV isolate T36 [83]. Comparison of the 2.4 Kbp CTV-VT sequence with the full-length T36 sequence confirmed close similarity but also revealed puzzling directional differences, which indicated that the 2.4 Kbp fragment represented a defective (d)RNA composed of the fused parts of the CTV genome 5′ and 3′ ends [84]. The finding of the first dRNA of CTV-VT explained the difficulties of cloning the full-length CTV-VT cDNA genome and led to the detection of additional dRNAs associated with CTV infections [85]. In later studies, parts of some dRNAs were found to correspond to subgenomic RNA regions, including the 3′ terminal CTV gene, *p23*, and its 3′ non-coding RNA sequence [86], suggesting their synthesis through two separate biosynthetic events, including the 3′ end subgenomic RNA and the full-length genomic RNA replication [87]. Other dRNAs included entire 5′ genomic halves with short 3′ termini [88] or the entire 3′ genomic halves with shortened 5′ ends [87], pointing to complex processes of the large CTV genome formation [89]. It is interesting to note that, except in some cases of certain mild stem pitting CTV-VT isolates associated with a CTV isolate carrying the large 5′ half dRNA [88] and a range of seedling yellows reactions [90], neither of which were firmly proved by firm genetic analyses, our understanding of the biological role of these abundantly present dsRNA molecules in CTV infections remains unknown [91].

Realizing the problem resulting from basing the CTV-VT cloning efforts on the genetic information derived from the 2.4 dRNA molecule, we changed the cDNA synthesis extension strategy. Within several months, we completed the sequencing of the CTV-VT genome, which became the second CTV isolate to be fully sequenced [92]. Sequence comparisons between the CTV-VT and T36 genomes revealed significant genomic divergence, particularly at the 5′ half of their genomes. Despite these substantial differences, we refrained from classifying them as separate species due to their similar genomic organization, replication strategy, and biological characteristics, including vector transmission and host range.

Further studies revealed that tissue infected with different CTV strains contained two positive-sense RNA molecules corresponding to the 5′ end of the CTV genome. The most abundant was short (~0.7 Kb), termed low-molecular-weight tristeza (LMT), and the second most abundant were the far larger positive RNA molecules, termed LaMT, representing the entire 5′ half of the CTV genomic region [93,94]. It is interesting to note that unlike the 3′ end CTV subgenomic RNAs, which possess both plus and minus strands, the two 5′-end subgenomic molecules consist only of plus-strand molecules, indicating that the CTV negative-strand genomic RNA serves as the template for their synthesis. The function of the LaMT remains unknown, while LMT continues to attract attention [95,96,97].

## 14. Transgenic Resistance

Pathogen-derived resistance to TMV was successfully achieved in transgenic plants expressing the virus coat protein gene [98]. Inspired by this promising result, our laboratory focused on developing transgenic citrus rootstocks and varieties resistant to CTV. Two PhD students, Drs. Dan Piestun and Ozgur Batuman, dedicated nearly a decade to this task. They produced transgenic citrus plants expressing various CTV genes. However, when tested under simulated field conditions, none of these plants showed transgenic protection against CTV [99,100].

The difficulty of publishing these failures posed significant challenges to their academic progress. The difficulty was further exacerbated by our unmet goal of developing desirable citrus rootstocks, such as *C. macrophylla*, with CTV resistance and by several publications from respected laboratories reporting more positive results.

In 2019, personal correspondence with Dr. Leandro Peña, whose group was conducting similar research, confirmed that their transgenic citrus plants with CTV constructs did not exhibit resistance under practical field conditions. This shared disappointment highlights the challenging research objective of understanding the barriers preventing effective transgenic resistance to CTV—and possibly to other *Closteroviridae* of horticultural importance.

Another high-priority challenge is, in my opinion, deciphering the genetic basis of sour orange resistance to CTV transmission by aphids [45]. This subject is of considerable importance for Mediterranean countries in the event of an *A. citricidus* invasion, as it is an efficient CTV vector. It is also critical for other regions should cross-protection fail.

## 15. Retirement Years

I retired officially after almost 40 years of service at ARO, Volcani Center, on 1 September 2004. However, my lab continued operating for two more years to support my last two students completing their thesis. Following retirement, I rented a small plot of land (about an acre) and planted a diverse collection of citrus and several other subtropical fruit trees. Unfortunately, a severe freeze in February 2008 damaged most of the trees—a harsh reminder of the challenges of making a living from horticulture.

Working in the grove, I experimented with seed grafting on citrus trees [101]. I used a procedure of intense callus formation on massively girdled juvenile trees to shorten the juvenility of citrus seedlings. Attempts to transform citrus trees in-grove failed due to difficulty inducing embryo formation of wound-induced callus. The grove provided ample fruit for family and friends, but its main function was to give me the pleasure of continued contact with citrus trees—an experience magnified during the recent COVID-19 pandemic.

Following retirement, I continued interacting with citrus virus researchers, including Dr. Dawson [95] and Dr. Svetlana Folimonova [73] in Florida and Catania [46]. The continued contact with past colleagues and former students, including Dr. Ozgur Batuman, who kindly provided editorial assistance on this manuscript, remains highly valued and deeply cherished.

## 16. Discussion

In an article in the *Citrus Industry Magazine*, the late Nancy G. Hardy began her report with the question, “What has changed in Floridian citrus?” Her response was simple: “Everything”. Hardy’s remark about changes in Florida’s citrus industry was made long before the emergence of the catastrophic citrus greening disease (Huanglongbing, or HLB) in 2005, which dramatically changed and reduced Florida’s citrus production. A similar statement could be made about citrus production in Israel during my service, albeit for different reasons—chiefly the lack of water, rising production costs, and High-Tech employment opportunities.

Looking back, I consider myself fortunate in several ways. My work on purifying CTV virions began as a gamble. Virus isolation, particularly without reliable methods to evaluate the product throughout the process, was both cumbersome and risky. In retrospect, I feel deeply indebted after years of disagreements with Prof. Gad Loebenstein, my late supervisor, who allowed me the freedom to conduct endless trials, even during nearly three years of failing results. The CTV virion isolation problem was resolved using PEG for virus precipitation. Later, at the JII, a modification of the CTV isolation method enabled the purification of BYV and facilitated the establishment of the expanding Closterovirus group. The abundance of dsRNAs in Closterovirus-infected plants remains a mystery. However, it has already benefited researchers by helping overcome the challenges of purifying the thread-like virions of several newly identified Closteroviruses, including the economically important grapevine leafroll viruses.

The pioneering sequencing and genetic analysis of the BYV genome by researchers of Agranovsky and Atabekov’s other former students in Russia and their German partners was a major milestone in Closteroviridae research [102,103] and the foundation for the genetic analysis of the first completely sequenced CTV strain, T-36, in Florida [81]. The research in Florida contributed to the discovery of multiple defective RNAs in CTV-infected tissue, the role of which remains unclear [91]. Sequencing of the CTV-VT strain revealed significant and unusual genetic variation among CTV strains [92]. Further research in Dr. Dawson’s lab led to the construction of an infectious clone of CTV [104], a major achievement that transformed CTV from a dangerous pathogen into a valuable genetic tool [5,105,106].

I consider myself fortunate to have devoted my entire research career primarily to a single plant virus problem of significant importance, with only a few diversions to other key pathogens. The local citrus industry supported my work at the Institute until 1986 in exchange for my contributions to the CTV suppression program and other practical efforts, such as disease-free budwood propagation. In later years, until my retirement in September 2004, I received partial funding from the Citrus Board for specific services, including the rapid release of virus-free budwood derived from the Institute’s citrus breeding program.

I am deeply grateful for the generous support of BARD and other national and international funding organizations, which enabled me to establish my laboratory. Named after the renowned citriculturist Mr. S. Tolkowsky [107], the lab was staffed by a technician and several graduate students, funded through competitive research grants. Securing sufficient funding to sustain the lab over the years was greatly aided for several years before retirement by my collaboration with Dr. W.O. (Bill) Dawson, who aptly compared the relentless search for funding to mountain climbing—where one hand clings to a ledge while the other searches for the next foothold.

Looking back on my “mountain climbing” journey, which began with many unknowns about citrus viruses, I can say that I have been “vectored” by CTV to numerous research institutes and citrus-producing regions worldwide. These experiences enriched my work through knowledge exchange and collaborations with researchers across the globe. I am indebted to many dear colleagues who generously shared their expertise, publications, and writing skills with me—an area in which I still strive to improve. While I cherish all of them, I particularly miss Drs. S.M. Garnsey and Ricardo Flores, dear friends with whom I exchanged emails almost weekly.

My deepest thanks and love go to Hanna and our children, Ziv, Chen, and Yaara—now parents themselves—who as children endured my long hours spent with ultracentrifuges, PAGE, and ELISA in my quest to uncover the mysteries of tristeza. Their patience, understanding, and unwavering support have been the foundation of my journey, and I am profoundly grateful for their sacrifices.

Reflecting on my work, it seems that advancements in practical disease control often have the unintended consequence of cutting the very branch on which we relied, at least from the perspective of the management of local citriculture. Nevertheless, I find satisfaction in the advancements achieved by my lab and their impact on both the industry and the scientific community. However, assessing the full scope of this impact requires comprehensive analyses that extend beyond individual perspectives.

I believe that research institutes should entrust independent evaluation committees with the task of objectively assessing the value of past efforts. Such evaluations would not only provide a clearer understanding of the outcomes of previous initiatives but also yield valuable insights to guide future investments in research and development. By fostering a culture of accountability and strategic planning, these measures could help ensure that scientific progress remains aligned with the evolving needs of industries and communities.
